# The complete chloroplast genome sequence of *Vitis amurensis* ‘Shuanghong’

**DOI:** 10.1080/23802359.2020.1780975

**Published:** 2020-06-22

**Authors:** Dinghan Guo, Dongmei Li, Ruiqi Wang, Bin Han, Shiren Song, Wenping Xu, Shiping Wang, Lei Wang, Jianxin Niu, Chao Ma

**Affiliations:** a Department of Horticulture, College of Agriculture, Shihezi University, Shihezi, Xinjiang, China; b Department of Plant Science, School of Agriculture and Biology, Shanghai Jiao Tong University, Shanghai, China; c Hebei Academy of Agricultural and Forestry Sciences, Fruit Research Institute, Changli, China; d YunNan (DaLi) Research Institute, Shanghai Jiao Tong University, YunNan, China

**Keywords:** *Vitis amurensis* ‘Shuanghong’, chloroplast genome, Illumina sequencing, phylogenetic analysis

## Abstract

*Vitis amurensis* ‘Shuanghong’ is a hybrid offspring of wild grapes. This study first releases the complete chloroplast genome of *V. amurensis* ‘Shuanghong’ and subjected the sample to phlogenetic analysis. The chloroplast genome is 161,558 bp in length, and comprises a small single-copy region (19,336 bp) and a large single-copy region (89,744 bp), which are seperated by a pair of inverted repeat regions. The chloroplast genome encodes 133 genes, including 88 CDSs, 8 rRNA genes, and 37 tRNA genes. The phylogenetic tree showed that *V. amurensis* ‘Shuanghong’ is most closely related to *Vitis vinifera*.

*Vitis amurensis* ‘Shuanghong’ shows extremely strong cold-resistant and downy mildew resistance properties. So, it is widely used for breeding of grapes against downy mildew (Zhao Y et al. 2020). It is also an excellent wine brewing variety because of high anthocyanins content (Zhao Q et al. 2016). The complete chloroplast genome of *V. amurensis* ‘Shuanghong’ was assembled (GenBank; MT479164) and subject to phlogenetic analysis. It offers useful information for the resistance breeding of grapevine.

Genomic DNA was extracted from leaves of *V. amurensis* ‘Shuanghong’ grown at the Modern Engineering Training Center (31°11′N, 121°29′W) and stored at the Center for Viticulture and Enology, Shanghai Jiao Tong University, number was “Shuanghong”. This DNA was used for the preparation of a 400 bp small-fragment DNA library and then sequenced using the HiSeq PE150 sequencing platform (Illumina, CA, USA). A total of 3.22 Gb clean reads were obtained. The complete chloroplast genome was assembled by A5-MiSeqv20150522 (Coil et al. 2015) and SPAdesv3.9.0 (Bankevich et al. 2012) software. The chloroplast genome of *V. vinifera* (GenBank; DQ424856) was used as a reference (Jansen et al. 2006) and the genome annotation also referred to the chloroplast genome of *V. vinifera*.

The *V. amurensis* ‘Shuanghong’ chloroplast genome is 161,558 bp in length, including two inverted repeat regions (26,239 bp each) that are separated by a small single-copy region (19,336 bp) and a large single-copy region (89,744 bp). The chloroplast genome contains 133 single genes, including 88 protein-coding genes (CDS), 8 rRNA, and 37 tRNA genes. The GC content and AT content of the grape genome is 36.58% and 63.42%, respectively. Among these genes, the majority are single copy, whereas 8 CDS (*rp12*, *rp123*, *rps7*, *rps12*, *rps19*, *ycf2*, *ycf15*, *ndhB*), 7 tRNAs (*trnI-CAU*, *trnL-CAA*, *trnV-GAC*, *trnI-GAU*, *trnA-UGC*, *trnR-ACG*, *trnN-GUU*) and 4 rRNAs (*rrn4.5*, *rrn5*, *rrn-16*, *rrn-23*) occur as double copies.

A neighbor-joining (NJ) phylogenetic tree was constructed using 16 *Vitis* species through the MEGA X (Kumar et al. 2018). To identify the phylogenetic position of *V. amurensis* ‘Shuanghong’ within the family Vitaceae, the phylogenetic tree showed that the 16 *Vitis* species are clustered into two orders (Figure 1). The *V. amurensis* ‘Shuanghong’ was phylogenetically closer to *Vitis vinifera* of Europen species than species in other generas.

**Figure 1.**
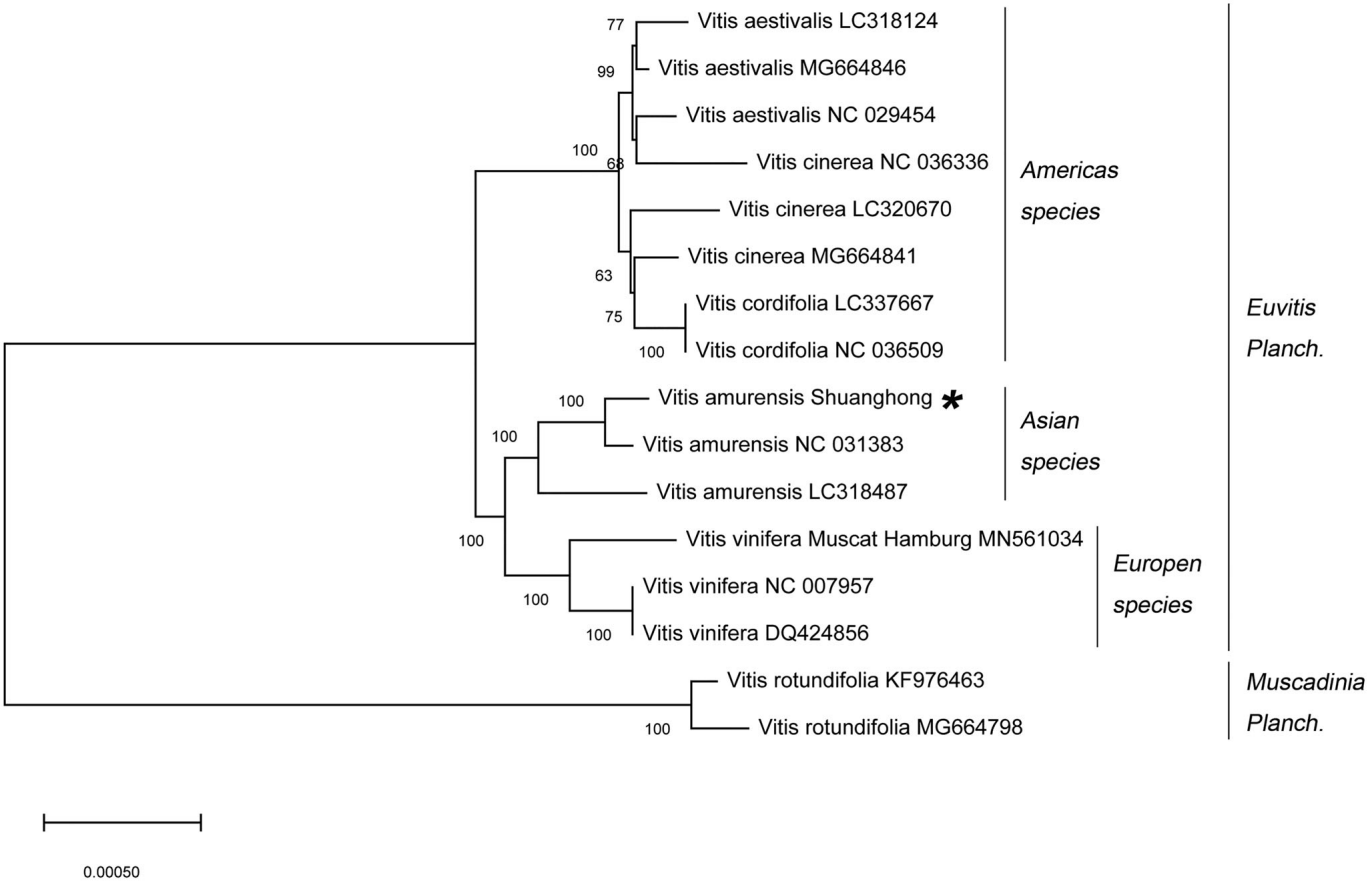
A neighbor-joining (NJ) phylogenetic tree was constructed by using 16 *Vitis* species. *Indicates *Vitis* variety in this study.

## Data Availability

The data that support the findings of this study are available in GenBank: MT479164 at https://www.ncbi.nlm.nih.gov/genbank/.

## References

[CIT0001] Bankevich A, Nurk S, Antipov D, Gurevich AA, Dvorkin M, Kulikov AS, Lesin VM, Nikolenko SI, Son P, Prjibelski AD, et al. 2012. SPAdes: a new genome assembly algorithm and its applications to single-cell sequencing. J Comput Biol. 19(5):455–477. 22506599 10.1089/cmb.2012.0021 PMC3342519

[CIT0002] Coil D, Jospin G, Darling AE. 2015. A5-miseq: an updated pipeline to assemble microbial genomes from Illumina MiSeq data. Bioinformatics. 31(4):587–589. 25338718 10.1093/bioinformatics/btu661

[CIT0003] Jansen RK, Kaittanis C, Saski C, Lee S-B, Tomkins J, Alverson AJ, Daniell H. 2006. Phylogenetic analyses of *Vitis* (Vitaceae) based on complete chloroplast genome sequences: effects of taxon sampling and phylogenetic methods on resolving relationships among rosids. BMC Evol Biol. 6(1):32. 16603088 10.1186/1471-2148-6-32 PMC1479384

[CIT0004] Kumar S, Stecher G, Li M, Knyaz C, Tamura K. 2018. MEGA X: molecular evolutionary genetics analysis across computing platforms. Mol Biol Evol. 35(6):1547–1549. 29722887 10.1093/molbev/msy096 PMC5967553

[CIT0005] Zhao Q, He F, Reeves MJ, Pan Q-H, Duan C-Q, Wang J. 2016. Expression of structural genes related to anthocyanin biosynthesis of *Vitis amurensis*. J Res. 27(3):647–657.

[CIT0006] Zhao Y, Wang Z-X, Yang Y-M, Liu H-S, Shi G-L, Ai J. 2020. Analysis of the cold tolerance and physiological response differences of amur grape (*Vitis amurensis*) germplasms during overwintering. Sci Hortic. 259:108760.

